# Assessment of Indian wheat germplasm for Septoria nodorum blotch and tan spot reveals new QTLs conferring resistance along with recessive alleles of *Tsn1* and *Snn3*

**DOI:** 10.3389/fpls.2023.1223959

**Published:** 2023-10-10

**Authors:** Sudhir Navathe, Xinyao He, Umesh Kamble, Manjeet Kumar, Madhu Patial, Gyanendra Singh, Gyanendra Pratap Singh, Arun Kumar Joshi, Pawan Kumar Singh

**Affiliations:** ^1^ Genetics and Plant Breeding Group, Agharkar Research Institute, Pune, India; ^2^ Global Wheat Program, International Maize and Wheat Improvement Centre (CIMMYT), Texcoco, Mexico; ^3^ Division of Crop Improvement, ICAR-Indian Institute of Wheat and Barley Research, Karnal, India; ^4^ Division of Genetics, ICAR-Indian Agricultural Research Institute, New Delhi, India; ^5^ Indian Council of Agricultural Research (ICAR)-National Bureau of Plant Genetic Resources, New Delhi, India; ^6^ International Maize and Wheat Improvement Centre (CIMMYT) & Borlaug Institute for South Asia (BISA), New Delhi, India

**Keywords:** *Parastagonospora nodorum*, *Pyrenophora tritici-repentis*, quantitative inheritance, *Triticum aestivum*, *Tsn1*

## Abstract

The leaf blight diseases, Septoria nodorum blotch (SNB), and tan spot (TS) are emerging due to changing climatic conditions in the northern parts of India. We screened 296 bread wheat cultivars released in India over the past 20 years for seedling resistance against SNB (three experiments) and TS (two experiments). According to a genome-wide association study, six QTLs on chromosome arms 1BL, 2AS, 5BL, and 6BL were particularly significant for SNB across all three years, of which *Q.CIM.snb.1BL, Q.CIM.snb.2AS1*, *Q.CIM.snb.2AS.2, and Q.CIM.snb.6BL* appeared novel. In contrast, those on 5BS and 5BL may correspond to *Snn3* and *Tsn1*, respectively. The allelic combination of *tsn1*/*snn3* conferred resistance to SNB, whereas that of *Tsn1*/*Snn3* conferred high susceptibility. As for TS, *Tsn1* was the only stably significant locus identified in this panel. Several varieties like PBW 771, DBW 277, and HD 3319, were identified as highly resistant to both diseases that can be used in future wheat improvement programs as resistant donors.

## 1 Introduction

Wheat (*Triticum* spp.) is a staple food and a crucial element of global food security. However, of several factors, fungal diseases are the most important that limit wheat production. Septoria nodorum blotch (SNB) is caused by the necrotrophic fungal pathogen *Parastagonospora nodorum* (syn. *Phaeosphaeria nodorum* [E. Müll.], syn. *Leptosphaeria nodorum* [E. Müll.], syn. *Stagonospora nodorum* [Berk.], syn. *Septoria nodorum* [Berk.]). SNB frequently co-occurs with other necrotrophic fungal diseases like tan spot (TS, caused by *Pyrenophora tritici-repentis*) and Septoria tritici blotch (STB, caused by *Zymoseptoria tritici*). This disease is common in areas that experience frequent or high rainfall during the wheat growing season, such as Australia, Canada, Scandinavia, Central and Eastern Europe, eastern USA, and South America (Bearchell et al., 2005; Solomon et al., 2006; Shaw et al., 2008). In India, the disease was first recorded in the Nilgiri hills of south India (Chona and Munjal, 1952) and later from the Kumaon hills in Northern India (Joshi et al., 1971). In the last few decades, it has been encountered frequently in the northwestern plains zone (NWPZ) of the country, especially during cool and wet Rabi seasons (Rana et al., 2000). Due to the changing climatic conditions, SNB has been expanding into new niches. For example, it was first observed on emmer wheat (*T. dicoccoides*) in Turkey in 2017 and has been reported recently in Himachal Pradesh, India (Cat et al., 2018; Katoch et al., 2019).

Tan spot (TS), or yellow leaf spot, is a serious wheat disease affecting temperate and tropical wheat-growing regions. The fungal pathogen, a necrotroph that causes minor to severe spotting in wheat, was first described in 1823 (Hosford, 1982). The disease was subsequently reported in Europe, the USA, and Japan in the early 1900s (Wegulo, 2011). In India, reports on TS infection from northern plains and central regions were documented between 1934 and 1972 (Mitra, 1934; Misra and Singh, 1972), followed by a more recent one in 2007 (Singh, 2007). The development of the disease is encouraged by the favourable climate in South Asia, particularly in the Himalayan and eastern Gangetic plains, where low temperature and humidity favour prolonged leaf wetness. It has been reported that TS co-occurs with spot blotch in some parts of Nepal, causing yield losses of up to 20-30% (Duveiller et al., 2005). In Nepal, there is a higher incidence of foliar blight in the last week of January to mid-February when the temperature is still cool, and a few genotypes were identified as tolerant to the disease, like NL750, Milan, and Shanghai-7 (Dubin and Bimb, 1994; Duveiller et al., 2005; Gurung et al., 2012). So far, no systematic work has been done on SNB and TS in India except for reports on its occurrence. Moreover, no isolates have been deposited in the national fungal culture collection and type culture collections.

It is thought that *P. nodorum* obtains nutrients from dying plant tissue caused by secreted effectors. These effectors cause host hypersensitivity and result in programmed cell death (Friesen et al., 2007; Oliver et al., 2012). So far, eight effectors (SnToxA, SnTox1, SnTox2, SnTox3, SnTox4, SnTox5, SnTox6, and SnTox7) have been identified to date, along with nine major wheat sensitivity loci that correspond to them: *Tsn1, Snn1, Snn2, Snn3-B1/Snn3-D1, Snn4, Snn5, Snn6*, and *Snn7*, respectively (Friesen et al., 2007; Friesen and Faris, 2012; Tan et al., 2012; Phan et al., 2016).

Marker-trait associations (MTAs) of polygenetic traits in plants have been widely identified using genome-wide association studies (GWAS). Adhikari et al. (2011); Gurung et al. (2014); Liu et al. (2015), and Phan et al. (2018) have all discovered MTAs for SNB resistance at seedling resistance. In seedling experiments for resistance against a *P. nodorum* isolate lacking SnTox3, QTL was found on 2D, 3A, and 5B (Adhikari et al., 2011; Gurung et al., 2014). The 5B QTL’s association with the *Tsn1* gene, which has been extensively studied (Liu et al., 2009; Gurung et al., 2014), supports the ability of GWAS to identify QTL for SNB resistance. TS resistance is both quantitatively and qualitatively inherited, where toxicity resistance genes and QTL have been identified. Tan spot resistance (Tsr) refers to the quality genes discovered through conidial inoculations, and “*Tsc*” and “*Tsn*” refer to genes for chlorosis and necrosis reactions against HST-containing cultures, respectively (McIntosh et al., 2008). In addition to the host susceptibility gene *Tsn1*, eight significant *Tsr* genes have so far been discovered, i.e., *Tsrl, Tsr2, Tsr3, Tsr4, Tsr5, Tsr6, TsrHar*, and *TsrAri*, being located on the chromosomes 2BS, 3AS, 3BL, 3DS, and 5BL (Kokhmetova et al., 2021; Lozano-Ramírez et al., 2022).

Though spot blotch remained a major concern in the Indian subcontinent among the foliar blotch diseases, it is important to do pre-emptive screening work on SNB and TS, considering the past reports and future outbreak risk under climate changing scenarios. In the present study, a panel of 296 bread wheat genotypes released in India over the past 20 years was screened at seedling stages for SNB and TS resistance. Further, the population was studied for the presence of the major toxin sensitivity gene *Tsn1*, and genome-wide association studies were performed for the genetic basis of the resistance.

## 2 Materials and methods

### 2.1 Plant material and genotyping

This study used a panel of 296 bread wheat genotypes from released varieties and advanced breeding lines developed primarily over the last twenty years from 25 research centres across India. DNA extraction and DArT sequencing of the genotypes were done per the protocol demonstrated by Li et al. (2016). The wheat accessions were sequenced with the DArTseq^®^ technology at the Genetic Analysis Service for Agriculture (SAGA) at CIMMYT, Mexico. Markers with a minor allele frequency of less than 10% (2804 markers) or more than 30% missing data points (96 markers) were excluded from further analysis. A total of 9668 SNPs were finally used for the GWAS analysis, and their physical positions on the reference whole genome sequence (IWGSC: Chinese Spring RefSeq v1.0, International Wheat Genome Sequencing Consortium et al., 2018) were acquired from the database https://wheat-urgi.versailles.inra.fr/). The SNP markers were given names based on their chromosome location followed by clone ID, e.g., 5BL:3955588.

### 2.2 Disease screening for SNB and TS

The panel was evaluated for 3 years (2019, 2021, 2022) for SNB and 2 years for TS (2019, 2021) in greenhouse at the seedling stage. Mexican *P. tritici-repentis* (Ptr) isolate *MexPtr1* and *P. nodorum* isolate *MexSn4* were used for resistance screening against TS and SNB, respectively. Both isolates are ToxA producers based on inoculation experiments using differential genotypes, infiltration experiments, and PCR with the ToxA-specific marker (data not shown). The isolates were grown on V8-PDA media (Lamari and Bernier, 1989), and conidiospore concentrations for inoculation were adjusted to 4 × 10^3^ spores mL^−1^ (*MexPtr1*) and 1 × 10^7^ spores mL−1 (*MexSn4*) (Singh et al., 2007; Singh et al., 2016). The response of TS and SNB was tested on seedlings in a greenhouse at 22°C day and 18°C night temperatures with a 16-h photoperiod. Experiments were set up in a randomized complete block design with two replicates, with four plants grown in plastic containers as experimental units to derive mean values for further analysis. Erik and Glenlea were used as resistant and susceptible controls, respectively. Inoculations were performed when the second leaf was fully expanded at around 14 days after planting. The inoculum was applied to seedlings with a hand sprayer until runoff (about 0.5 mL inoculum per plant). The trays were transferred to a humid chamber (RH 100%, 20°C) once the leaves were dry to promote infection, and the plants were returned to the greenhouse bench after 24 hours. Both diseases were rated on a linear scale of 1–5 at seven days after inoculation (dpi) (Feng et al., 2004; Hu et al., 2019).

### 2.3 Linkage disequilibrium and population structure

All 9668 SNP markers were used to calculate a kinship matrix, clusters among individual genotypes, and a heat map using the traditional Van Raden (2008) equation using TASSEL v5 (http://www.maizegenetics.net, accessed on 25 Oct 2022). Linkage disequilibrium (LD) was analysed with R^2^ among SNP markers plotted against the physical distances in mega base pairs (Mb) across the 21 wheat chromosomes. The genotypic data was numerically transformed for population structure analysis utilizing XLSTAT (v. 2022.1). Structure 2.3.4 software was used to obtain the population structure (Pritchard et al., 2000). The admixture model was adjusted with a 100,000 burn-in period followed by 500,000 marker chain Monte Carlo (MCMC) iterations. The subpopulation test range was maintained at K1 - K5, with five iterations. The actual subpopulations were assessed using the DK approach (Earl and vonHoldt, 2012), which was verified using the Structure Harvester program (Web v0.6.94, Earl and vonHoldt, 2012) as per the method described by Evanno et al. (2005). The output summary calculated the standard deviation and average logarithm of the probability of the observed likelihood [LnP(D)]. The log-likelihood of the data was computed for each class (K = 1 to 5) to determine LnP(D) for each MCMC step. A neighbour-joining tree was created using TASSEL 5.0 (Bradbury et al., 2007) and visualized using the iTOL website (Letunic and Bork, 2021).

### 2.4 Genome-wide association analysis

For GWAS, three models (MLM, MLMM and FarmCPU) implemented in the R package GAPIT3 (Wang and Zhang, 2021) were used. The first was the mixed linear model (MLM=K+Q), which was based on the kinship matrix (K) and the principal component (PC) (Yu et al., 2006). The second method was the Multiple Loci Mixed Linear Model (MLMM), which employs forward-backward stepwise linear mixed-model regression to include associated markers as covariates. In contrast to MLM and MLMM, the third model FarmCPU (fixed and random, circulating probability unification); used all linked markers in a fixed-effect model and optimised the linked markers in a separate random-effect model, reducing false positives and false negatives and enabling quick computation. Population structure, principal components, and kinship were used as covariates in MLM and MLMM, and their QQ plots were compared. Our phenotypic data fit the FarmCPU model better, and QTL showed higher significance; therefore, only data analyzed by the FarmCPU model was chosen for additional examination. The Bonferroni correction (α=0.1) was used for an exploratory significance threshold to uncover putative QTL (Chan et al., 2010). QTL was recognized as robust when linked markers reached the strict -log10(p) criteria of 3.0 in at least being detected across-year by either threshold adopted in this investigation and consistent across the models. The Quantile-Quantile (QQ) plots were also examined to determine the point at which the observed p-values diverge from those predicted by the null hypothesis. SNP marker annotations were obtained using the databases http://www.cerealsdb.uk.net and https://triticeaetoolbox.org. The physical positions of markers on the reference whole genome sequence (IWGSC: Chinese spring RefSeq v1.0, International Wheat Genome Sequencing Consortium et al., 2018) were acquired from the database https://wheat-urgi.versailles.inra.fr/ and https://plants.ensembl.org/Triticum_aestivum/Info/Index. If two significant markers shared a 10-Mbp interval or had substantial LD (R^2^>0.8) with one another, they were regarded as belonging to the same QTL region.

### 2.5 KASP genotyping for *Tsn1*

Kompetitive Allele-Specific PCR (KASP) marker Ktsn1 specific for *Tsn1* (Dreisigacker et al., 2016) was used for genotyping the panel. Genomic DNA was isolated from 21-day-old seedling leaves, DNA concentration was measured with a Nanodrop 8000 spectrophotometer (Thermo Scientific), and the final concentration of 10 ng/ml was obtained by diluting the DNA with sterile PCR-grade water. KASP genotyping was undertaken using the PACE master mix per the manufacturer’s guidelines (3CR biosciences). In brief, the assay mix for 25 samples was prepared as 3µl VIC primer, 7.5 µl common primer, and 14.5 µl of PCR grade H2O. Finally, for reaction assembly, individual reactions contained 5 µl 2X PACE, 0.138 µl assay mix, and 5 µl genomic DNA. The samples were run on a CFX96 Real-time PCR system (Bio-Rad). The PCR steps included 94°C for 15 min, 10 cycles of touch down program (94°C-0.20 min, 65 to 57°C-1 min), followed by 30 cycles of (94°C-0.20 min, 57°C-1 min). The results were analyzed in Bio-Rad CFX manager 3.1, and output was obtained as.csv files. The results obtained were compared against the linked SNP calls from the SNP array.

### 2.6 Haplotype analysis for the stable markers

For haplotype analysis, stable MTAs on chromosomes 1B, 2A, 5B, and 6B across experiments were selected. Eight markers that fitted the requirement that they were over the -log10(p) 3.0 threshold across the environments were chosen for the haplotype analysis: 5BS:1102120, 2AS:7487614, 2AS:1094287, 5BL:5324846, 5BL:3955588, 6B:1085698, 1B:1129298, and 5BL: Ktsn1. For TS, three markers, 5BL:5324846, 5BL:3955588, and 5BL : Ktsn1 were selected for the analysis. The corrected disease severities between haplotypes were compared using the Wilcoxon test implemented in R package ggpubr (Kassambara, 2020). The phenotypic data available for the seedling resistance to SNB in 2019, 2021, 2022, and TS in 2019 and 2021 was used for the analysis.

### 2.7 Stacking resistance allele and assessing interaction effect of *Tsn1* and *Snn3*

Data for three years (2019, 2021, and 2022) were used for this analysis. The corrected mean disease index from BLUP was used for haplotype analysis. Marker trait associations for SNB and TS were chosen from various models (MLM, MLMM, FarmCPU) to investigate the effect of stacking resistance alleles (**Table S1**). Resistant alleles were determined by mean comparison of corrected disease severity between alleles based on the Wilcoxon test, using the R package ‘*ggpubr*’ (Kassambara, 2020). Wheat lines were classified according to the number of resistant alleles present. The t-test (p < 0.05) was implemented in the R package ‘*multcomView*’ (Graves et al., 2015) to differentiate the significant groups.

To assess the gene effect of *Tsn1* and *Snn3*, two makers, 5BL:5324846 and 5BL:3955588, linked to *Tsn1* and data obtained from KASP assay Ktsn1 was compared with SNP 5BS:1102120 linked with *Snn3*. The interaction was discovered by comparing the mean between alleles using the t-test in the R package ‘*ggpubr*’ (Kassambara, 2020).

### 2.8 Statistical analysis

The corrected disease index, genotypic and phenotypic variance, and heritability estimates were obtained from META-R software (Alvarado et al., 2020). Additionally, mean comparisons with the Wilcoxon test, t-test, and data visualization were executed using packages `*ggpubr*`, ‘*dplyr*’, and ‘*multcomView*’ using R software (Version 4.2.1, R Core Team, 2022).

## 3 Results

### 3.1 Disease evaluations

The population displayed significant phenotypic variation for SNB and TS resistance with a skewed distribution towards the lower disease in all experiments (**Figure S1**). A high proportion of genotypes exhibited high resistance to both diseases (See **Table S1** for the disease scores) and the top 20 entries resistant to both diseases are presented in **Table 1**, including PBW 277, PBW 771, and HD 3319.

**Table 1:** List of wheat genotypes showing good resistance to Septoria nodorum blotch (SNB) and tan spot (TS), and their *Tsn1/tsn1* allele status.

No.	Genotype	Pedigree	SNB	TS	*Tsn1/tsn1*
1	PBW 771	BW9246/2*DBW17	1	1	*tsn1*
2	DBW 277	NI 5439/MACS 2496	1	1	*tsn1*
3	HD3319	18^th^ HRWYT214/18^th^ HRWYT229	1	1	*tsn1*
4	WH1256	SHA7//PRL/VEE#6/3/FASAN/4/HAAS8446/2*FASAN/5/CBRD/KAUZ/6/MILAN/AMSEL/7/FRET2*2/KUKUNA/8/2*WHEAR/SOKOLL	1	1	*Tsn1*
5	WH1258	CROC_1/Ae. Squarrosa (210)//WBLL1*2/BRAMBLING/3/VILLA JUAREZ F2009/5/BAV92//IRENA/KAUZ/3/HUITES*2/4/MURGA	1	1	*tsn1*
6	K1803	K 922/2K21	1	1	*tsn1*
7	K1805	K 922/2K21	1	1	*tsn1*
8	GW519	GW 394/PBW 519//AKAW 4627	1	1	*Tsn1*
9	TL 2969	JNIT-141/TL-1210//JNIT-141	1	1	*tsn1*
10	DBW93	WHEAR/TUKURU//WHEAR	1	1	*tsn1*
11	PBW802	HD2967*2/BWL3278	1.1	1	*tsn1*
12	UAS3002	RAJ4083/DWR195//HI 977	1.2	1	*tsn1*
13	UAS 3001	UAS259/GW322//HI 977	1.2	1	*tsn1*
14	UP3032	KAUZ//ALTAR84/AOS/3/MILAN/KAUZ/4/HUITES/UP2778	1.2	1	*tsn1*
15	MP3514	35IBWSN 244/DBW-17	1.3	1	*tsn1*
16	DBW285	PBW 550/SW89-5422	1.5	1	*Tsn1*
17	HD2733	ATTILA/3/HUITLE(HUI)/(CARC)CARCOMUN//CHEN/(CHTO)CHORLITO/4/ATTILA	1.5	1	*tsn1*
18	DBW 189	KACHU#1/4/CROC_1/Ae. Squarrosa (205)//BORL95/3/2*MILAN/5/KACHU	1.9	1	*tsn1*
19	DBW 168	SUNSU/CHIBIA	2	1	*tsn1*
20	DBW 273	FRANCOLIN #1*2//ND 643/2* WBLLI	1	1.1	*tsn1*
	Eric (Check)	Kitt//Waldron/Era	0	0	*tsn1*
	Glenlea (Check)	Sonora 64/Tezanos Pintos Precoz//Nainari 60	5	5	*Tsn1*

Analysis of variance revealed significant effects on genotype and genotype-by-environment for both diseases (**Table 2**). SNB showed higher overall heritability (0.87) than TS (0.75), with heritability in individual experiments ranging from 0.90 to 0.96.

**Table 2:** Basic statistics for Septoria nodorum blotch (SNB) and tan spot (TS) and analysis of variance across the environments.

Statistic	Overall BLUP_Tan_Spot	Overall BLUP_SNB	BLUP_Tan_Spot 2019	BLUP_Tan_Spot 2021	BLUP_SNB 2019	BLUP_SNB 2021	BLUP_SNB 2022
Heritability	0.759906126	0.872762979	0.909059	0.920974941	0.939572	0.938598	0.967649
Genotype Variance	0.580347871	0.680425265	0.883009	0.849503872	0.836392	1.169902	0.770685
Env. Variance	0.157381034	0.022362639	-	-	-	-	-
Residual Variance	0.161262888	0.104236798	0.176669	0.145784844	0.107584	0.153066	0.051531
Grand Mean	2.154363684	1.830977394	2.436716	1.872596656	1.874347	1.95768	1.657822
LSD	1.043675952	0.824142849	0.787204	0.722395105	0.624506	0.744523	NA
CV	18.64009824	17.63305443	17.24945	20.38975204	17.4994	19.98472	13.69298
Genotype significance	2.03917E-34	4.66E-106	-	-	-	-	-
Gen × Env significance	1.54425E-59	9.35E-128	-	-	-	-	-

### 3.2 Population structure and linkage disequilibrium analysis

**Figure 1:**
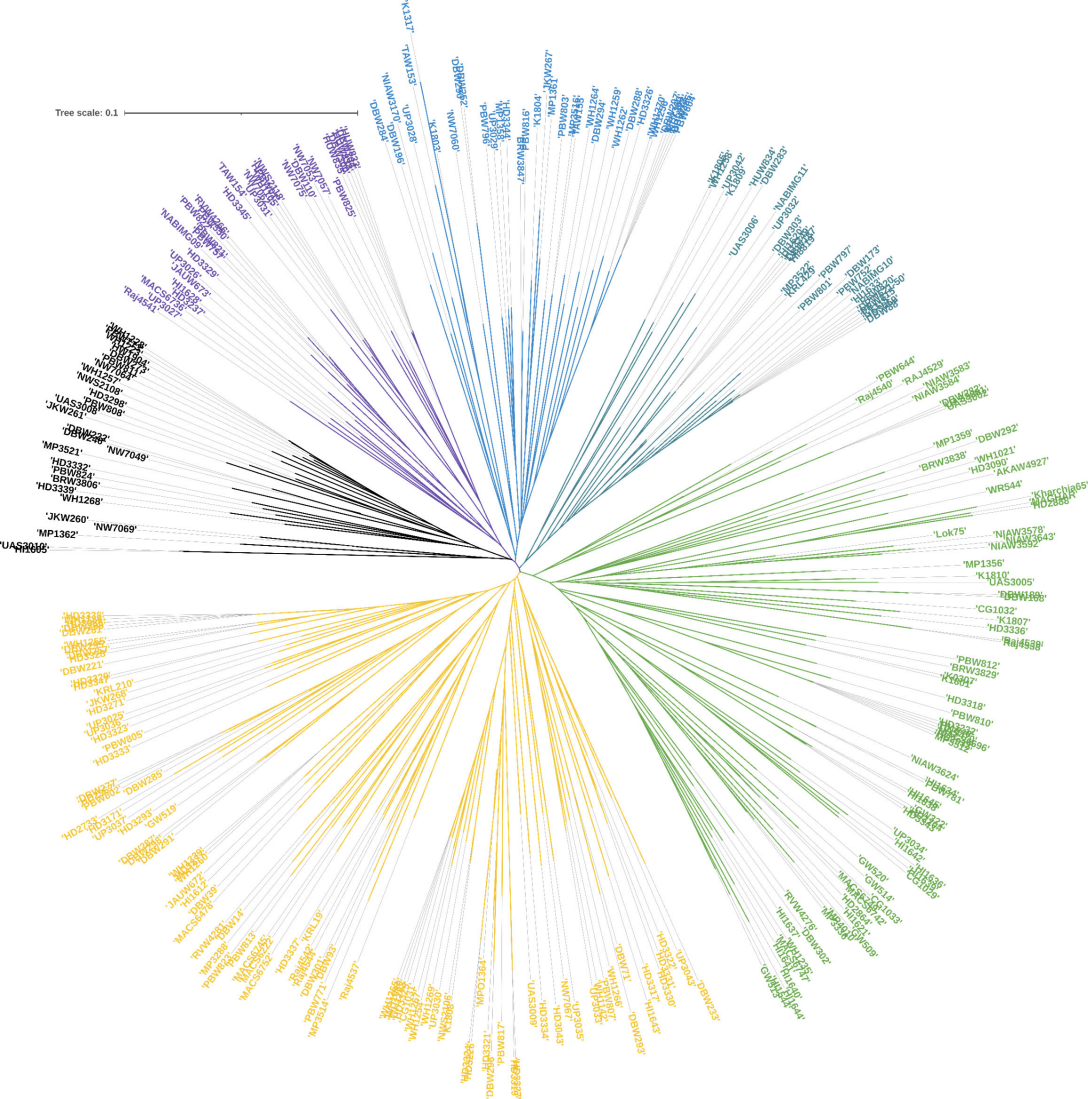
Population structures of 296 genotypes revealed by STRUCTURE 2.3.4 and neighbour-joining tree.

**Figure 2:**
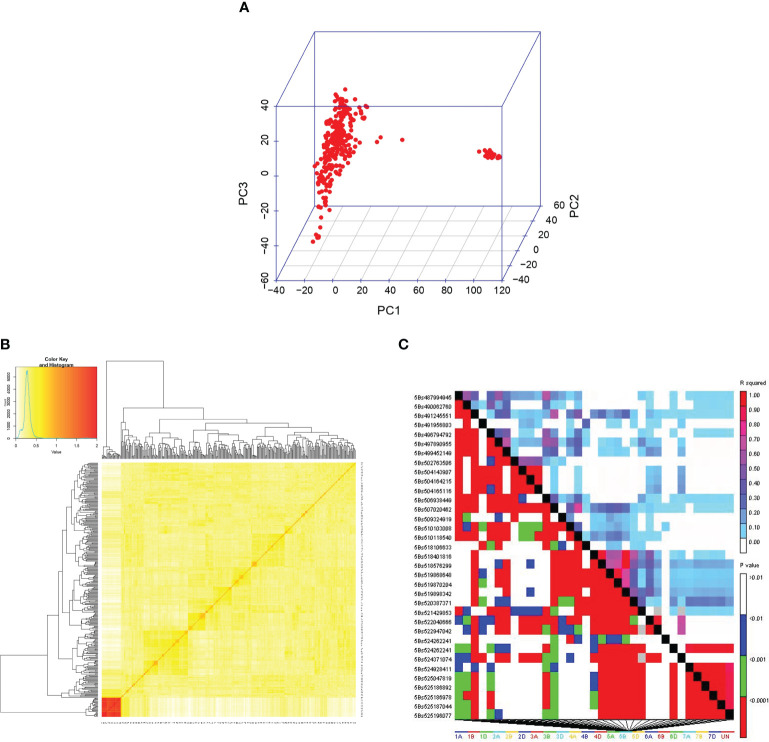
**(A)** 3D plots of principal components (PC). **(B)** The kinship matrix used in GWAS is visualized through a heat map. **(C)** LD matrix obtained from TASSEL 5.0.

Among the 9668 SNP markers selected for GWAS in the 296 bread wheat genotypes, 21.78% were from the A genome, 28.63% from the B genome, 12.03% from the D genome and 21.33% from unknown chromosomes. Population structure analysis based on the K means cluster approach divided the population into 4 major clusters that were depicted as a neighbour-joining tree (**Figure 1**). The kinship analysis also distinguished the population into 4 major groups and presented it as a kinship matrix-based heat map (**Figure 2**). The average extent of LD considered physical distance taken for the decay of R2 to reach a critical value of 0.10 across the genome, was approximately 10 Mb (**Figures 2**, **S2, S3**).

### 3.3 Genome-wide association study for SNB and TS

**Figure 3:**
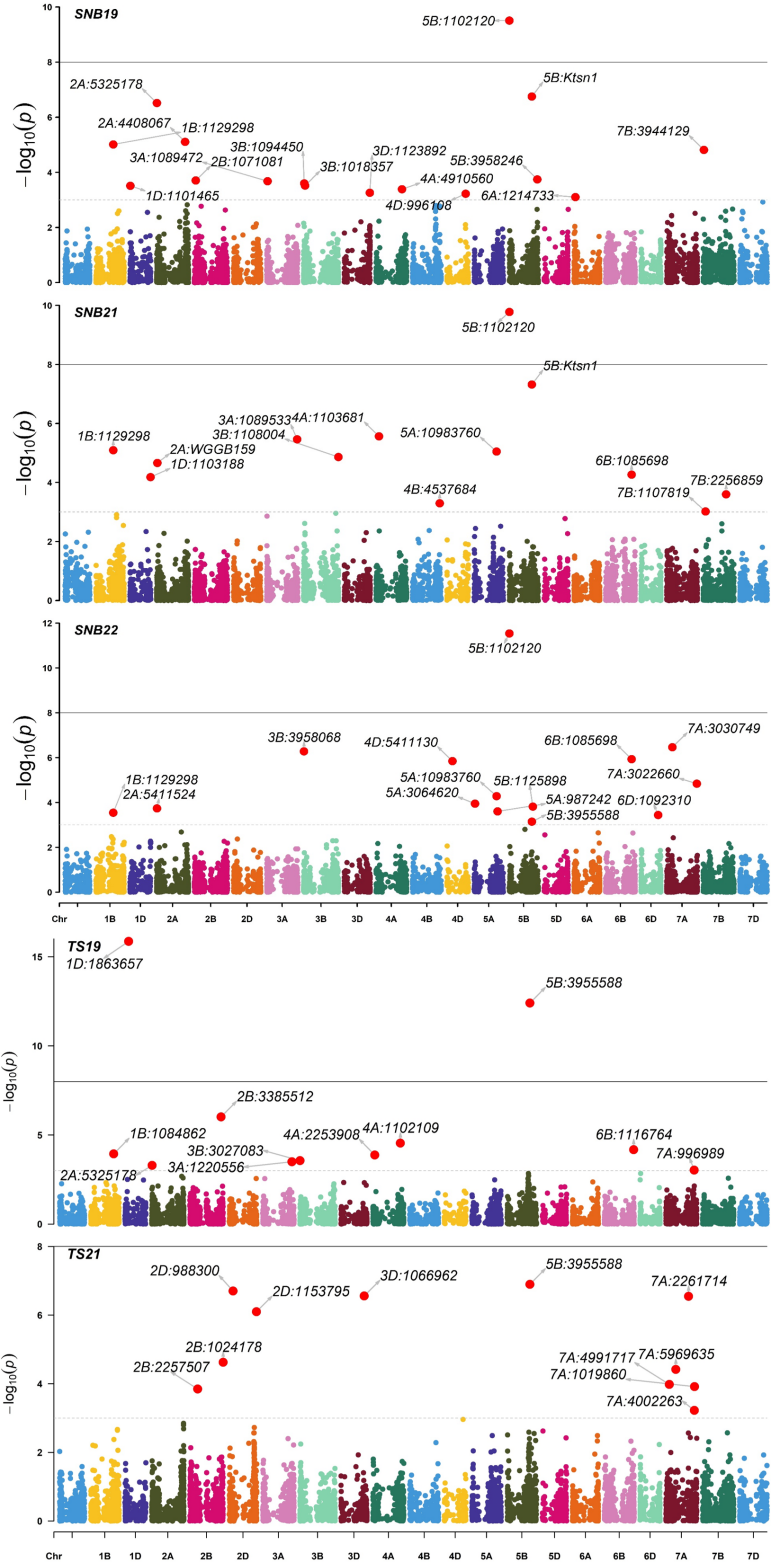
Manhattan plots of marker-trait associations detected by the FarmCPU model using 9668 single nucleotide polymorphisms in 296 Indian spring wheat genotypes evaluated for the seedling resistance to Septoria nodorum blotch (SNB) during years 2019, 2021, 2022 and tan spot (TS) during years 2019, 2021. The horizontal line represents the significant threshold –log10(P) = 5.00.

The exploratory -log10(p) threshold for the panel ranged from 2.92 to 11.53 for SNB and 2.96 to 15.86 for TS (**Tables S1**, **S2**). In total, 49 marker-trait associations (MTAs) were detected on various chromosomes for SNB (**Table S1**; **Figures 3**, **S4**). Six QTL on chromosomes 1BL, 2AS, 5BL, and 6BL were particularly significant for SNB over all three years and were considered robust QTLs. The QTL *Q.CIM.snb.1BL* on chromosome 1BL was identified with FarmCPU, and its peak marker 1BL:1129298 (*p* = 2.86E-04 to 8.97E-06) was located at 450.50Mbp on 1BL (**Table 3**). Two QTLs on 2AS, *Q.CIM.snb.2AS1 and Q.CIM.snb.2AS.2*, were detected in all models with their peak markers being 2AS:7487614 (59.43Mbp) and 2AS:1094287 (88.18Mbp), respectively. Another robust QTL, *Q.CIM.snb.5BS*, was identified at 5BS:1102120 (p=2.31E-05 to 5.99E-12) and was associated with *Snn3-B1*. Two *Tsn1*-associated markers 5BL:5324846 and 5BL:3955588 were detected across years and models on chromosome 5B at 566.04 and 588.4 Mbp, respectively (**Table 3**). The QTL *Q.CIM.snb.6BL* (p*=* 4.16E-04 to 1.61E-09) was detected using farmCPU, on the long arm of the chromosome 6B with peak marker 6BL:1085698 (669.13 Mbp).

**Table 3:** Markers significantly associated with Septoria nodorum blotch (SNB) and tan spot (TS) resistance through genome-wide association mapping using a mixed linear model (MLM), multi-locus mixed model (MLMM) and fixed and random model circulating probability unification (FarmCPU).

QTL/Susceptibility locus	SNP	Chr	Position	P.value	maf	effect	Experiments	Significant models
*Tsn1*	5BL:5324846	5BL	566048753	2.02E-04 to 1.96E-05	0.3078	0.329 to 0.362	SNB19, SNB21, SNB22	MLM, MLMM, FarmCPU
*Tsn1*	5BL:3955588	5BL	584471793	3.80E-04 to 5.43E-09	0.2781	-0.166 to -0.299	SNB19, SNB21, SNB22	MLM, MLMM, FarmCPU
*Snn3*	5BS:1102120	5BS	6008757	2.31E-05 to 5.99E-12	0.3816	0.246 to 0.365	SNB19, SNB21, SNB22	MLM, MLMM, FarmCPU
*Q.CIM.snb.2AS.1*	2AS:7487614	2AS	59436678	8.21E-04 to 9.39E-04	0.1277	0.271 to 0.358	SNB19, SNB21, SNB22	MLM, MLMM, FarmCPU
*Q.CIM.snb.2AS.2*	2AS:1094287	2AS	88181233	1.13E-03 to 9.06E-04	0.4049	-0.258 to -0.320	SNB19, SNB21, SNB22	MLM, MLMM, FarmCPU
*Q.CIM.snb.6BL*	6BL:1085698	6BL	669137486	4.16E-04 to 1.61E-09	0.2133	0.207 to 0.333	SNB21, SNB22	FarmCPU
*Q.CIM.snb.1BL*	1BS:1129298	1BS	450506896	2.86E-04 to 8.97E-06	0.1339	0.232 to 0.309	SNB19, SNB21, SNB22	FarmCPU
*Tsn1*	5BL:5324846	5BL	566048753	3.51E-05 to 5.41E-06	0.3078	0.376 to 0.410	TS19, TS21	MLM, MLMM, FarmCPU
*Tsn1*	5BL:3955588	5BL	584471793	1.33E-05 to 3.63E-13	0.2781	-0.258 to -0.397	TS19, TS21	MLM, MLMM

For TS, 79 MTA (-log10(p) >3) were detected on various chromosomes (**Table S2**; **Figure 3**). Two *Tsn1*-associated markers 5BL:5324846 and 5BL:3955588 were significantly detected across years. The SNP 5BL:5324846 (p=3.51E-05 to 5.41E-06) was detected across the three models (MLM, MLMM, FarmCPU). While the SNP 5BL:3955588 (p=1.33E-05 to 3.63E-13) was detected with a negative effect across the years (2019, 2021) from models MLM and MLMM only (**Table 3**).

Out of the 49 MTA identified for SNB resistance, 15 were selected to analyse their association with predicted genes and the corresponding biological functions. The criteria for shortlisting MTAs was that they were detected in at least two models or two experiments (See **Table 4**). Two *Tsn1*-associated markers, 5BL:5324846 and 5BL:3955588, were found to be associated with the transcripts *TraesCS5B02G409000*, *TraesCS5B02G387500*, and *TraesCS5B02G387000*. These transcripts are known to possess biological function pathogen stress response (PADRE) domain, Serine/threonine-protein kinase and Ubiquitin-like proteins respectively. Another marker, 2AS:7487614, has been found to be associated with two genes, namely *TraesCS2A02G107100* and *TraesCS2A02G107200*. These genes are known to have biological roles as a potassium transporter and a plant peroxidase, respectively. As for other MTAs, several noteworthy biological functions have been identified, including cysteine-type endopeptidase inhibitor activity (6B:1085698), NAD(P)-binding domain (4A:1082366), Cytochrome c oxidase subunit 5c, and Serine/threonine-protein kinase, active site (both for 3B:2251334). These functions are believed to play an active role in disease resistance (**Table 4**).

**Table 4:** SNPs associated with SNB and TS resistance and possible function elucidated based on the gene annotation using wheat reference sequence (RefSeq V1.0).

Sr	SNP	Position	P.value	Experiment	Model	Genomic region	Transcript	Biological function
**1**	5B:3955588	584471793	3.80E-04 to 5.43E-09	SNB19, SNB21, SNB22	FarmCPU, MLM, MLMM	5B:584289532-584472557	TraesCS5B02G409000	IPR025322: Pathogen and abiotic stress response, cadmium tolerance, disordered region-containing (PADRE) domain
						5B: 566675237-566683367	TraesCS5B02G387500	IPR008271:Serine/threonine-protein kinase, active site, IPR001611:Leucine-rich repeat, IPR000719:Protein kinase domain
**2**	5B:5324846	566048753	2.02E-04 to 1.96E-05	SNB19, SNB22	MLM, MLMM	5B:566047792-566128622	TraesCS5B02G387000	IPR000626: Ubiquitin-like proteins
**3**	5B: 1102120	6008757	2.31E-05 to 5.99E-12	SNB19, SNB21, SNB22	FarmCPU, MLM, MLMM	5B:6006645-6224311	TraesCS5B02G004200	PTHR13523: Coiled coil helix domain containing 2/NUR77
**4**	2A:1094287	88181233	1.13E-03 to 9.06E-04	SNB21, SNB22	MLM, MLMM	2A:88116755-88181997	TraesCS2A02G143000	IPR013094:Alpha/beta hydrolase fold-3
**5**	2A:7487614	59436678	8.21E-04 to 9.39E-04	SNB21, SNB22	FarmCPU, MLM, MLMM	2A:59435139-59594492	TraesCS2A02G107100	IPR003855: Potassium transporter
							TraesCS2A02G107200	IPR000823: Plant peroxidase
**6**	6B:1085698	669137486	4.16E-04 to 1.61E-09	SNB21, SNB22	FarmCPU, MLM, MLMM	6B:669049914-669139405	TraesCS6B02G394200	IPR027214: cysteine-type endopeptidase inhibitor activity
**7**	1B:1129298	450506896	2.86E-04 to 8.97E-06	SNB19, SNB21, SNB 22	FarmCPU	1B:450449347-450507660	TraesCS1B02G255700	IPR039620: BKI1/Probable membrane-associated kinase regulator 1/3/4
**8**	4D:5411130	143306414	1.42E-06 to 4.05E-04	SNB22	FarmCPU, MLM, MLMM	4D:143303907-143585661	TraesCS4D02G147900	IPR002464: DNA/RNA helicase, ATP-dependent, DEAH-box type, conserved site
**9**	4A:1082366	82791100	8.54E-04	SNB21	MLM, MLMM	4A:82691100-82891100	TraesCS4A02G080300	IPR036291: NAD(P)-binding domain
**10**	5B:1092387	622950551	3.77E-04	SNB21	MLM, MLMM	5B:622947949-622988941	TraesCS5B02G451500	IPR009577:Putative small multi-drug export
**11**	5B:1093198	569332682	9.52E-04	SNB22	MLM, MLMM	5B:569240491-569341529	TraesCS5B02G390100	IPR012876: Protein of unknown function DUF1677, plant
**12**	5A:10983760	576671605	5.20E-05 to 2.74E-04	SNB22	FarmCPU, MLM, MLMM	5A:576592693-576675256	TraesCS5A02G379400	IPR011141: Polyketide synthase, type III, IPR001099:Chalcone/stilbene synthase, N-terminal
**13**	5B:17335879	584572621	8.06E-05	SNB22	MLM, MLMM	5B:584569928-584622467	TraesCS5B02G409100	IPR025757: Ternary complex factor MIP1, leucine-zipper
**14**	3B:2251334	71642456	4.85E-04	SNB22	MLM, MLMM	3B:71639185-71816436	TraesCS3B02G105900	IPR008432: Cytochrome c oxidase subunit 5c
							TraesCS3B02G106100	IPR017441: Protein kinase, ATP binding site; IPR008271: Serine/threonine-protein kinase, active site
**15**	7B:3029515	652894544	6.68E-04	SNB21	MLM, MLMM	7B:652891171-652981239	TraesCS7B02G386800	IPR036574: Knottin, scorpion toxin-like superfamily

### 3.4 The significant role of *Tsn1* and *Snn3* on SNB resistance

**Figure 4:**
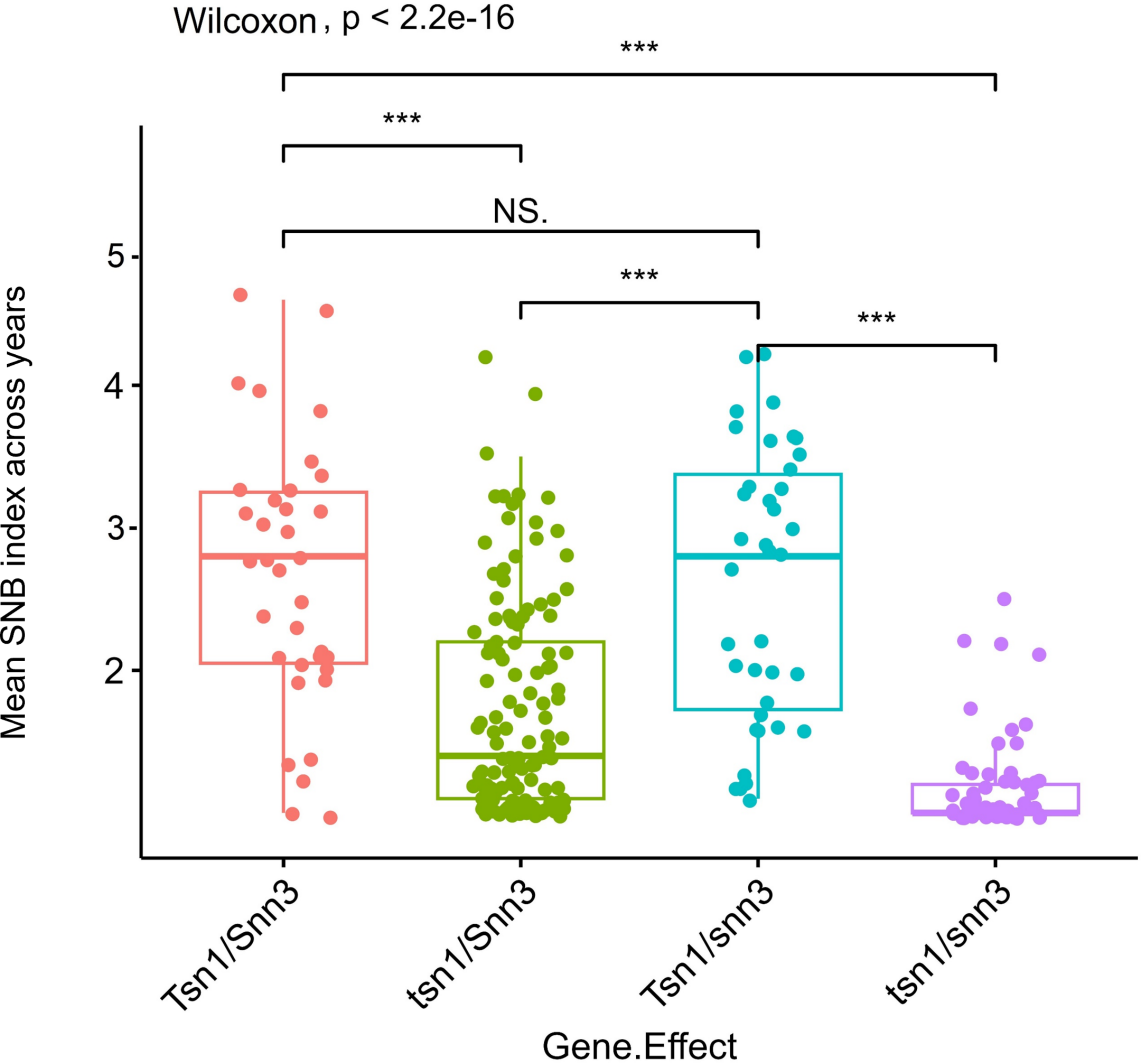
Boxplots showing gene interaction effect for *Tsn1* and *Snn3* in response to Septoria nodorum blotch (SNB). *** on boxplots indicates a significant difference in the mean disease index between groups by Wilcoxon test (p < 0.05).

The KASP assay revealed that 83 genotypes (25.85%) carry the susceptible allele *Tsn1*, whose SNB indices ranged mostly between 2.4 to 4.2 (**Table S3**). The allelic combinations at *Tsn1* and *Snn3* were tested using the results obtained from the *Ktsn1* marker and two *Tsn1* linked markers, 5B:5324846 and 5B:3955588, along with *Snn3-B1* linked marker 5BS:1102120. The comparison revealed that the allelic combination of recessive alleles *tsn1/snn3* imparts best resistance to SNB, followed by the *tsn1/Snn3* combination, whereas the allele combinations *Tsn1/snn3* and *Tsn1/Snn3* showed similar levels of resistance, indicating the higher phenotypic effects of *tsn1*(**Figure 4**).

### 3.5 Haplotype analysis and staking of R alleles

**Figure 5:**
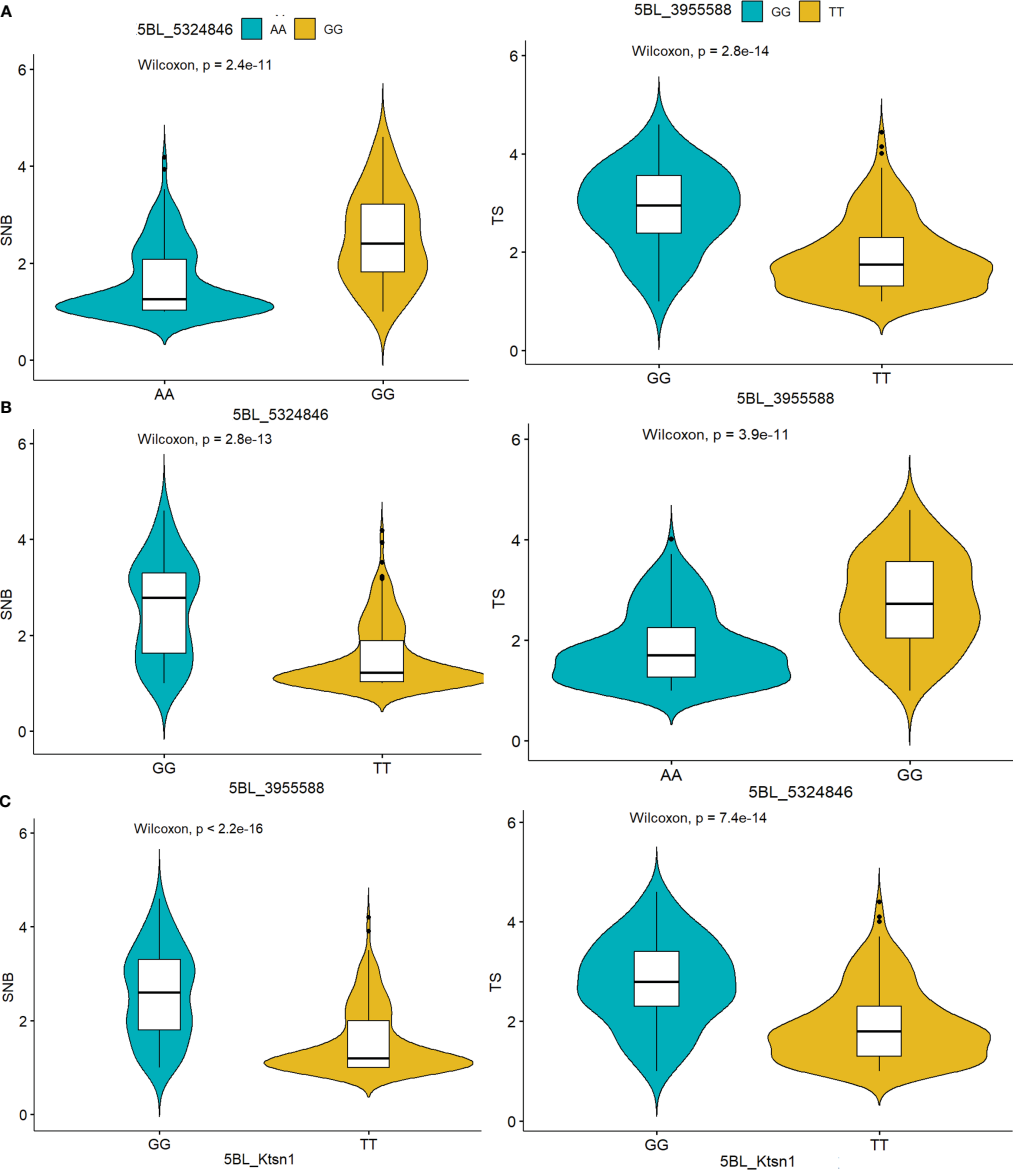
Haplotype analysis of the markers **(A)** 5BL:5324846 **(B)** 5BL:3955588 **(C)** KASP marker Ktsn1 indicated their significant association with Septoria nodorum blotch (SNB) and tans spot (TS) resistance determined by the Wilcoxon test.

There were significant differences in the corrected SNB index between resistant and susceptible haplotypes. When compared to the resistance haplotypes “AA/TT” for *tsn1* (mean SNB index 1.69), the susceptibility haplotype “GG” for *Tsn1* had a higher disease index (3.4) (**Figure 5**). Furthermore, the haplotypes were tested for the four significant SNB QTLs, *Q.CIM.snb.1BL, Q.CIM.snb.2AS.1, Q.CIM.snb.2AS.2*, and *Q.CIM.snb.6BL* (**Figure S5**). Similarly, there was a significant difference between susceptible and resistant haplotypes for TS index (3.2 for the susceptibility haplotypes and 1.57 for the resistance haplotypes; **Figure 5**).

**Figure 6:**
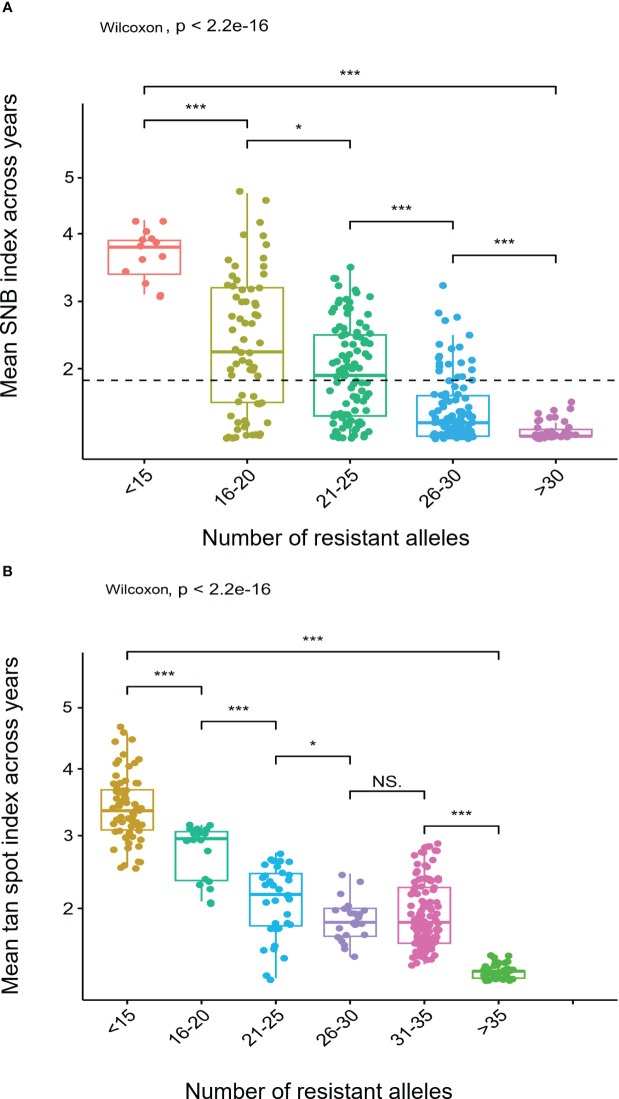
Boxplots showing effects of stacking resistant alleles for **(A)** Septoria nodorum blotch (SNB) and **(B)** Tan spot (TS). *** on boxplots indicates a significant difference in the mean disease index between groups by Wilcoxon test (p < 0.05).

The stacking of resistance alleles for SNB and TS exhibited a clear trend that the number of resistance alleles is positively correlated with disease resistance (**Figure 6**). For example, cultivars DBW 242 and DBW 246, each possessing 35 resistant alleles, exhibited very high SNB resistance, whereas HD 2932 and Kharchiya 65 having the least number of resistance alleles were highly susceptible. A similar trend was observed for TS resistance.

## 4 Discussion

Utilizing resistant cultivars coupled with management approaches contribute to environmentally and economically sustainable wheat production. The discovery of host-specific gene-for-gene interactions determining the *P. nodorum*-wheat pathosystem and residual genetic resistance (Liu et al., 2004) offers immediate opportunities to further investigate host-genetic resistance in wheat breeding (Ruud and Lillemo, 2018; Peters Haugrud et al., 2022). Evaluations of the Indian wheat cultivars reveal at least 113 genotypes resistant for the TS, 174 genotypes for SNB, and 118 for both the SNB and TS. Out of the top 20 highly resistant genotypes, WH1256, GW 519 and DBW 285 carry the susceptibility allele *Tsn1*, implying that they must have other resistance genes/QTL to counteract the negative effects of *Tsn1*. By checking the pedigree information, it was found that WH1256 and DBW 285 have Chinese progenitors and might have inherited unknown resistance genes.

We found seedling resistance QTL *Q.CIM.snb.2AS* for SNB close to previously reported QTL *QSnb.nmbu-2AS* (4-24 Mbp), which may be associated with two adult-plant leaf blotch QTLs at 2-20 and 15-16 Mbp on the reference genome (Lin et al., 2022). Additionally, this region was also mapped near the seedling resistance QTLs *Snb.niab-2A.1* (0.8-2.4 Mbp) and *Qsnb.cur-2AS2* (2.3-3.8 Mbp) reported by Lin et al. (2020) and Phan et al. (2016), respectively. *Snn3*-B1 has been associated with the short arm of chromosome 5B in previous research (Phan et al., 2016; Ruud et al., 2017; Ruud et al., 2019; Lin et al., 2020). Here, genetic analysis in the Indian wheat cultivars identified one marker 5BS:1102120 tightly linked to *Snn3*-B1 as representing the most significant genetic determinant of SnTox3 sensitivity in Indian germplasm.

Numerous shared QTLs between SNB and TS have been found in an increasing number of publications on the QTL mapping of both diseases (Phuke et al., 2020). A well-known example is *Tsn1* which confers sensitivity to both SnToxA and PtrToxA (Friesen et al., 2006). The current study has proven that *Tsn1* plays a major role in the susceptibility of the Indian panel to SNB and TS. TS resistance in seedlings and adult plants has also been discovered to be significantly influenced by the *P. nodorum* resistance/sensitivity QTL *Qsnb.cur-2AS.1* (Phan et al., 2016), discovered at the seedling and adult plant stage (Phan et al., 2016; Shankar et al., 2017). This phenomenon might suggest similar susceptibility/resistance mechanisms between the two diseases (Lin and Lillemo, 2021; Lin et al., 2022). It would be intriguing to learn if they share any additional effectors. To breed wheat varieties resistant to both diseases, wheat breeders can focus on mutual interactions, particularly for those QTL with relatively large effects and at both the seedling and adult stages.

The primary factor causing leaf necrosis is the HST ToxA, produced by the pathogens *P. nodorum* and *P. tritici-repentis* (produced by races 1, 2, 7, and 8). These pathogens are highly susceptible due to the sensitive gene *Tsn1*. The most prevalent Ptr race, Race 1, can also be found in South Asia and Mexico (Duveiller and Singh, 2009). However, *P. nodorum* from South Asia lacks information on the racial composition, variability, and toxins produced. In the last 25 years, South Asian breeding programs have incorporated new knowledge about resistance to leaf blight disease complex. Researchers and breeders worldwide could ascertain the relationship between effector sensitivity and cultivar susceptibility using expressed ToxA since 2005 and Tox3 since 2011. The *ToxA* sequence in various *B. sorokiniana* isolates from India has been analyzed recently, and the results show that the gene is under positive selection (Navathe et al., 2020). The investigations on the pathogenicity factors, variability, and toxin profiling of *P. nodorum* and *P. tritici-repentis* from South Asia is lacking, irrespective of their numerous reports from India and Nepal. The horizontal transfer of *ToxA* between these three pathogens underlines that unless *Tsn1/Snn3* is selectively bred out of widely planted wheat germplasms, it is likely that ToxA will continuously evolve into forms that are more effective in inducing host cell death. Other cutting-edge technologies will accelerate the discovery and functional characterization of effector resistance genes in the coming years and offer effective methods for utilizing these in breeding programs.

India and Nepal have reported SNB, TS, and spot blotch occurrences individually or in the complex. Moreover, climate change has warranted cross-continent jump in wheat diseases and we know very little about the interactions between these three diseases. Further, this subject is especially fascinating given that *P. nodorum, P. tritici-repentis* and *B. sorokiniana* share effectors. Because a new cultivar must be fully assessed at adult plant stage in field conditions, breeding for SNB/TS/SB resistance has always been difficult. The difficulties are made worse by inoculation using a representative group of isolates. Making significant annual isolate collections, especially from the cultivar that is currently most resistant, is one clear recommendation that has come out of recent studies. These novel isolates can be examined phenotypically for novel effectors and virulence traits and genotypically to follow specific chromosomal regions. Any new effector can be expressed, and their contribution to virulence can be evaluated. The main benefit of isolate collections is that they make it possible to rationally choose the smallest set that accurately captures all the pathogen’s phenotypic diversity, against which resistance should be sought.

At present, complete genetic resistance to SNB and TS has not been discovered. Therefore, a multifaceted strategy based on agronomic practices, disease surveillance, and genetic resistance will be required for the effective management of SNB and TS. Nevertheless, genetic resistance will continue to play a major role in managing these diseases. It was interesting to see that some varieties like PBW 771, DBW 277, and HD 3319 displayed high levels of resistance to both diseases. Hence, they must be deployed in the ongoing breeding programs for further enhancement of resistance for SNB and TS. Using field resistance and knowledge about new QTLs will certainly help breeders find much better resistance for these diseases facilitating higher production in the farmer’s fields.

## Data availability statement

The original contributions presented in the study are included in the article/supplementary material. The genotypic data is available at Dataverse CIMMYT data repository and can be accessed at https://hdl.handle.net/11529/10548934. Further inquiries can be directed to the corresponding author/s.

## Author contributions

SN contributed to manuscript writing and data analysis, XH contributed to conceptualization, disease scoring and data analysis, and UK, MK, and MP helped in data analysis and manuscript writing. PKS and AJ contributed to the conceptualization, project monitoring, fund acquisition and revising. GPS and GS contributed to germplasm resources and fund acquisition. All authors contributed to the article and approved the submitted version.
