# Supplementary material for: Assessment of Indian wheat germplasm for Septoria nodorum blotch and tan spot reveals new QTLs conferring resistance along with recessive alleles of Tsn1 and Snn3
Source: Front Plant Sci. 2023 Oct 10;14:1223959. doi: 10.3389/fpls.2023.1223959 (PMC10597639; doi:10.3389/fpls.2023.1223959)
Supplement: Supplementary file 1 [file Presentation_1.pptx]

## Slide 1
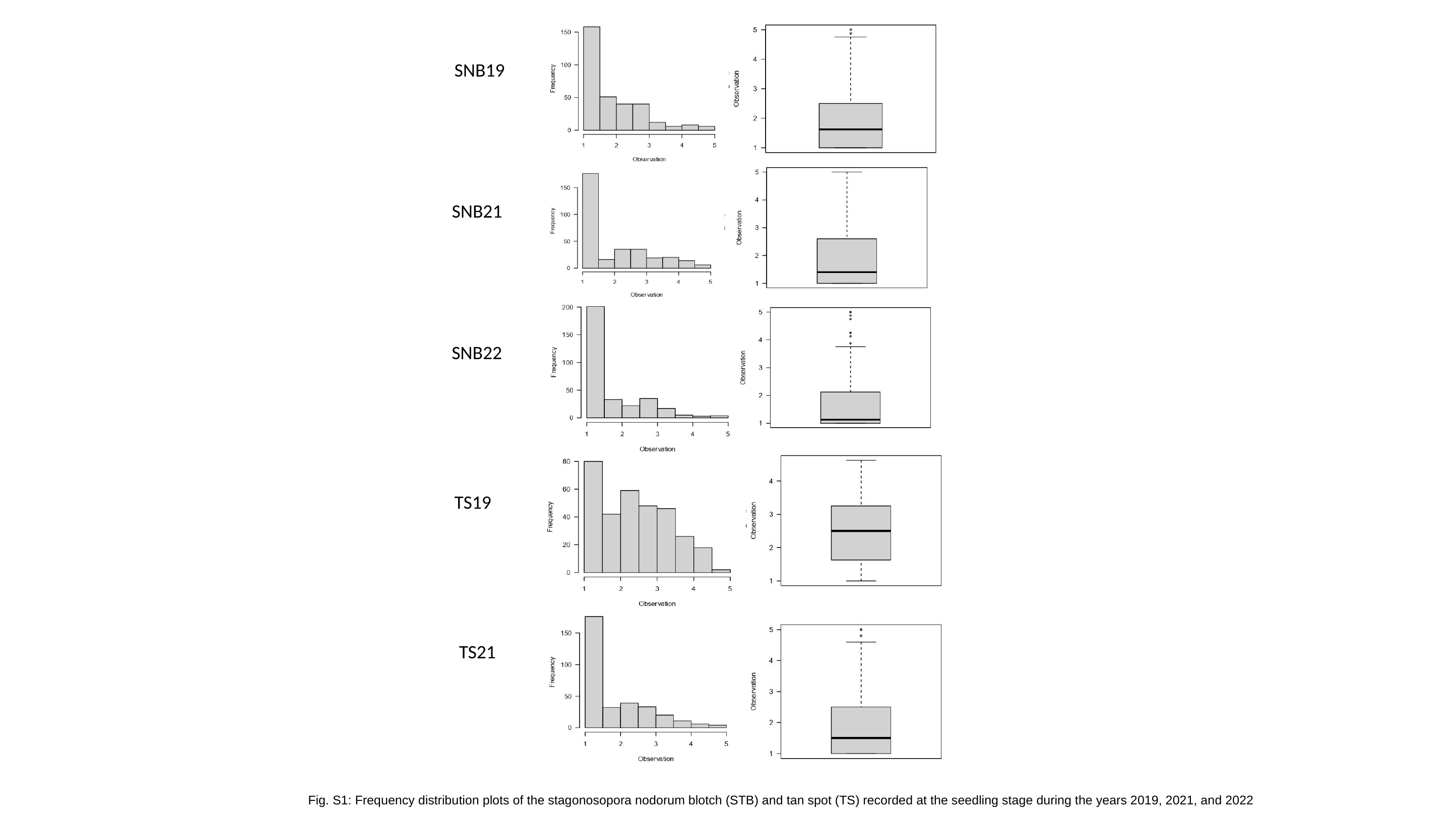

SNB19
SNB21
SNB22
TS19
TS21
Fig. S1: Frequency distribution plots of the stagonosopora nodorum blotch (STB) and tan spot (TS) recorded at the seedling stage during the years 2019, 2021, and 2022

## Slide 2
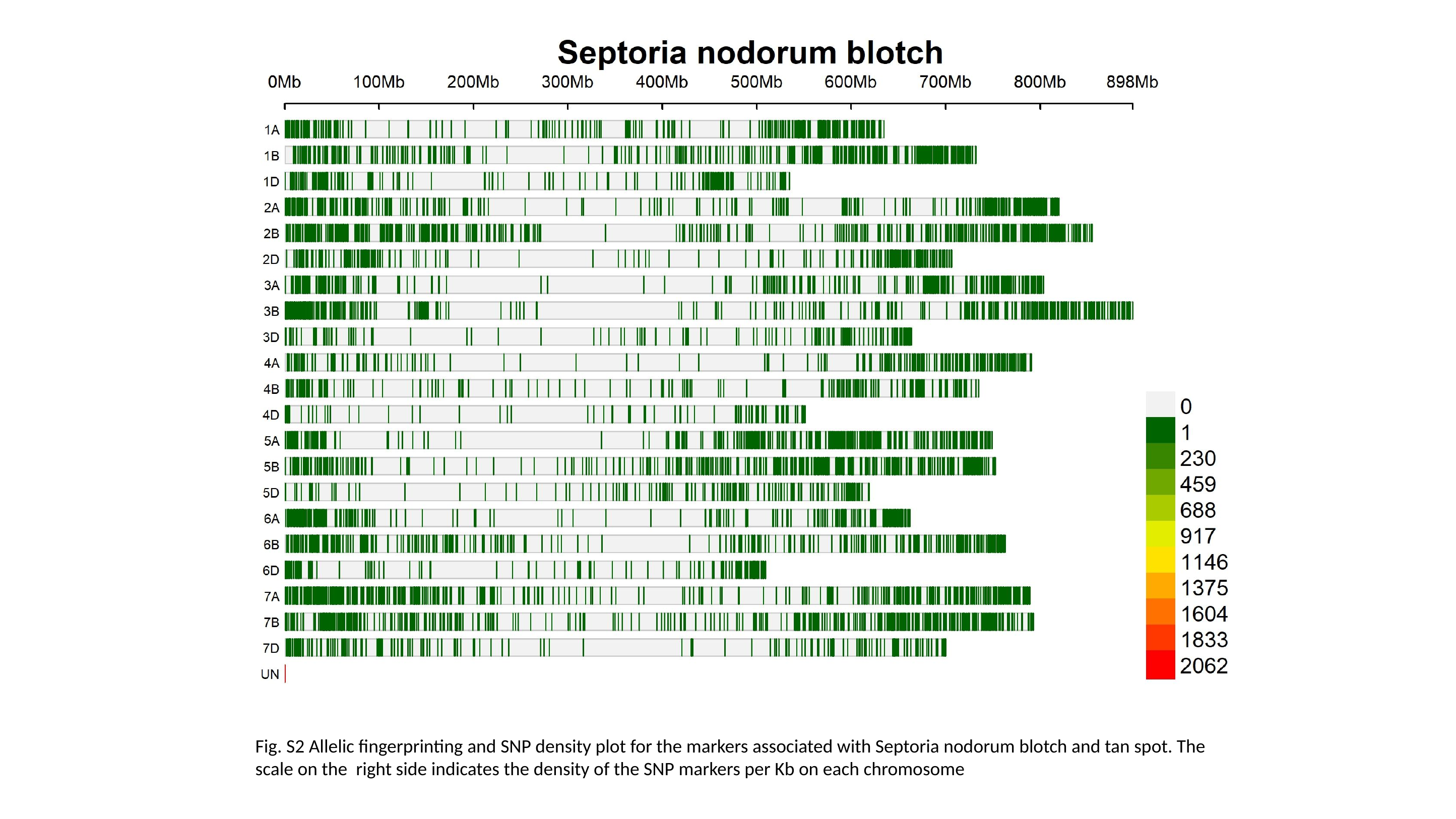

Fig. S2 Allelic fingerprinting and SNP density plot for the markers associated with Septoria nodorum blotch and tan spot. The scale on the right side indicates the density of the SNP markers per Kb on each chromosome

## Slide 3
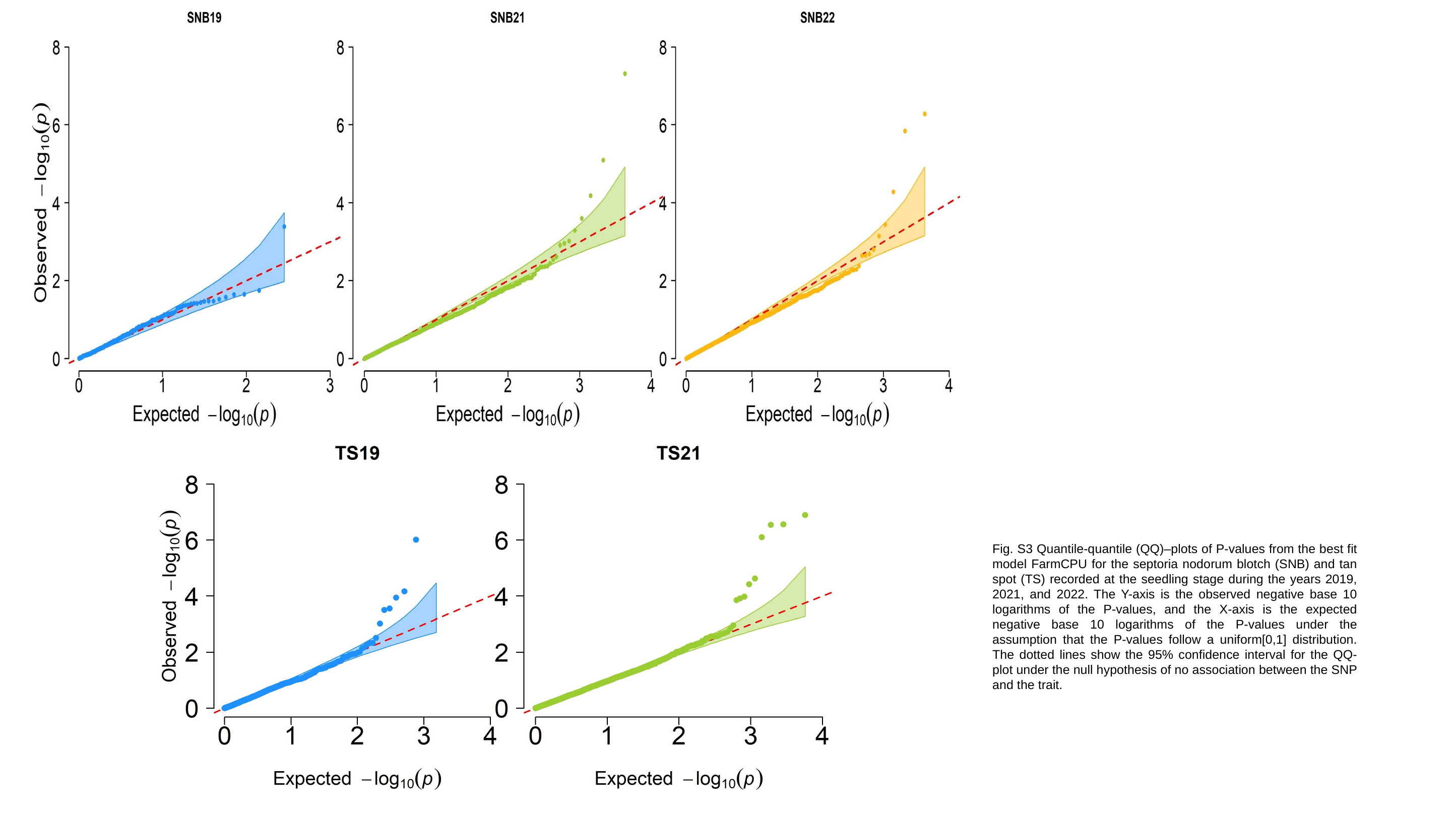

Fig. S3 Quantile-quantile (QQ)–plots of P-values from the best fit model FarmCPU for the septoria nodorum blotch (SNB) and tan spot (TS) recorded at the seedling stage during the years 2019, 2021, and 2022. The Y-axis is the observed negative base 10 logarithms of the P-values, and the X-axis is the expected negative base 10 logarithms of the P-values under the assumption that the P-values follow a uniform[0,1] distribution. The dotted lines show the 95% confidence interval for the QQ-plot under the null hypothesis of no association between the SNP and the trait.

## Slide 4
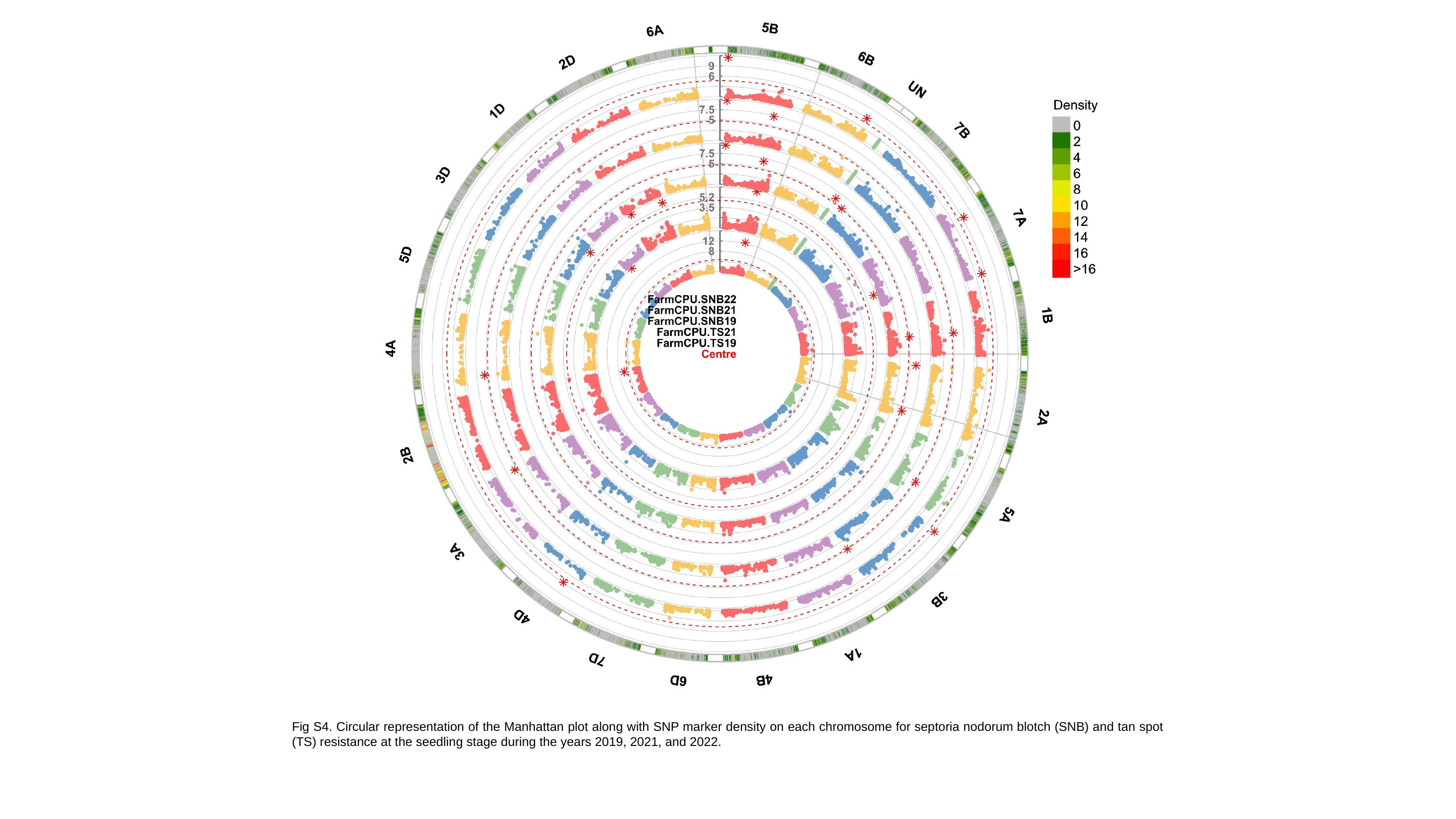

Fig S4. Circular representation of the Manhattan plot along with SNP marker density on each chromosome for septoria nodorum blotch (SNB) and tan spot (TS) resistance at the seedling stage during the years 2019, 2021, and 2022.

## Slide 5
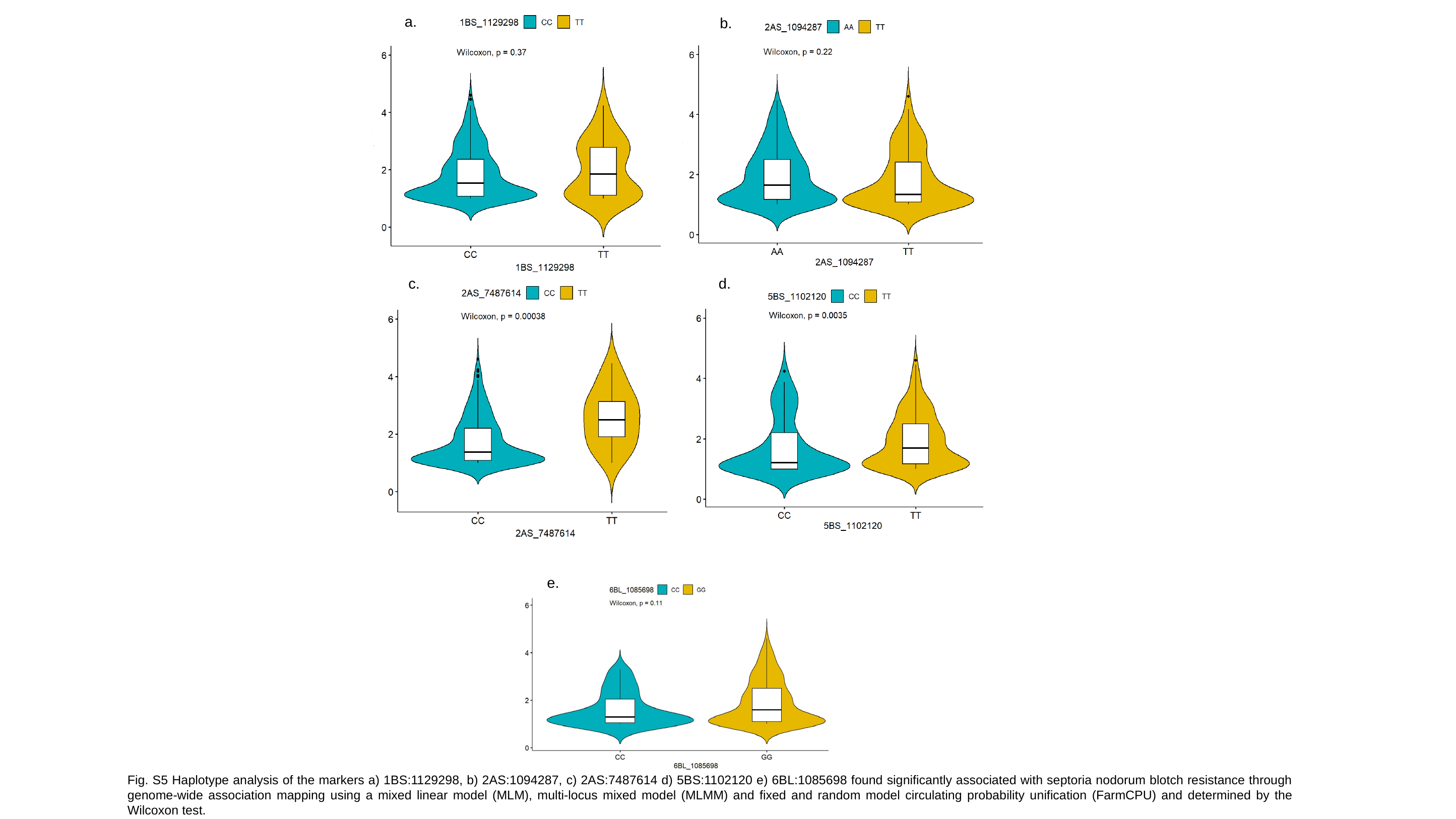

a.
b.
c.
d.
e.
Fig. S5 Haplotype analysis of the markers a) 1BS:1129298, b) 2AS:1094287, c) 2AS:7487614 d) 5BS:1102120 e) 6BL:1085698 found significantly associated with septoria nodorum blotch resistance through genome-wide association mapping using a mixed linear model (MLM), multi-locus mixed model (MLMM) and fixed and random model circulating probability unification (FarmCPU) and determined by the Wilcoxon test.
